# Quantitative determination of mercury in blood-based DL-cysteine-modified direct mercury analyzer method

**DOI:** 10.3389/fbioe.2026.1776977

**Published:** 2026-03-12

**Authors:** Zuoxiang Li, Teng Hou, Wenqiao Long, Bo Chen, Ling Li, Yi Li, Haoyu Zhou, Yi Zhu

**Affiliations:** Department of Clinical Laboratory, The Third People’s Hospital of Guizhou Province, Guizhou, Guiyang, China

**Keywords:** blood mercury, direct mercury analyzer, DL-cysteine, ethylmercury, methylmercury

## Abstract

Occupational mercury poisoning is a significant work-related illness due to that mercury vapor and its compounds can enter the human body and cause systemic damage. Therefore, regular blood mercury monitoring is essential for occupationally exposed individuals. Among existing detection methods, mercury analyzers are widely used for direct blood mercury determination. Despite the utility of direct mercury analyser (DMA), their application to blood samples is often compromised by limitations such as method instability and mercury carryover. To address these challenges, this study introduces a DL-cysteine-assisted determination method. It is based on the principle that DL-cysteine forms stable complexes with mercury in blood, thereby mitigating container adsorption and memory effects. Specifically, DL-cysteine solution was incorporated into both the mercury calibration standards and the blood samples within the sample boat prior to analysis using a DMA. The limit of detection was 0.5 μg/L, and the limit of quantification was 1.5 μg/L. Method accuracy, evaluated by spiked recovery, ranged from 99.14% to 99.92%. Experiments demonstrated that the stability, recovery, and repeatability of the direct mercury determination method were improved by introducing DL-cysteine, which offers practical value in enhancing the performance of direct mercury determination methods and is suitable for routine blood mercury monitoring.

## 1 Introduction

Mercury is a naturally abundant element and a potentially toxic industrial metal, which is frequently released into the environment via drinking water, dietary intake, and industrial activities (Charkiewicz et al., 2025; Li et al., 2024; Rahman et al., 2023). In environmental media, mercury exists in various forms, including inorganic elemental mercury, monovalent mercury, divalent mercury, and organic forms such as methylmercury (MeHg) (Hong et al., 2015). The toxicity of mercury is closely linked to its chemical speciation. For example, compared to other forms, Hg^2+^ and MeHg exhibit higher affinity for thiol-containing proteins and enzymes, resulting in more pronounced toxic effects (Wang et al., 2023). Due to its unique chemical properties and inherent toxicity, mercury can inflict severe damage on the internal organs of the human body upon ingestion and absorption (Henriques et al., 2025; Cronin et al., 2005; Song et al., 2022; Strähle et al., 2020). Mercury poisoning has become a highly debated global public health concern; consequently, timely and facile detection of mercury in biological samples is critical for clinical diagnosis.

Given the metabolic behavior of mercury in the human body, the selection of appropriate sample types for exposure monitoring is essential to accurately reflect exposure levels and corresponding temporal windows (Carrillo-Carrionet al., 2014; Wu et al., 2020; Jiang et al., 2017; Aaseth et al., 2020; Teschke, 2022). Blood mercury levels can rapidly reflect short-term mercury intake and exposure, and with a relatively short half-life in the blood (approximately 2–4 days), they serve as a reliable indicator of recent mercury exposure (within 1–2 weeks). Consequently, the blood sample is particularly valuable for the diagnosis of acute mercury poisoning, as well as an essential reference for assessing the incidence and severity of such recent exposure (Rocha et al., 2019; Björn et al., 2023; Moors et al., 2010; Karst et al., 2024; Janczak et al., 2024; Waara et al., 2007; McSorley et al., 2022; Haraguchi et al., 2018).

No unified standard has been established for the determination of mercury in whole blood to data. The primary methods for detecting mercury in blood include Inductively Coupled Plasma Mass Spectrometry (ICP-MS), Atomic Fluorescence Spectrometry (AFS), and Atomic Absorption Spectrometry (AAS) (Rutter et al., 2023; Musil et al., 2022). Among these, ICP-MS provides high sensitivity; when coupled with liquid chromatography, it enables speciation analysis of organic mercury and direct detection of various organic mercury species. Nonetheless, this method requires expensive instrumentation and involves relatively complicated operations. Similar to ICP-MS, AFS also offers high sensitivity. However, its measurement range is relatively narrow, and the procedure is comparatively complex (Luo et al., 2023). Therefore, a simple, rapid method requiring minimal sample pretreatment is required for the determination of mercury in blood.

The Direct Mercury Analyser (DMA) is a specialized instrument designed for measuring total mercury in blood. Its core detection principle involves converting all forms of mercury into gaseous atomic mercury via high-temperature pyrolysis and reduction for subsequent analysis: specifically, for blood samples, the instrument first reduces mercury in the sample to elemental mercury vapor through oxygen and a mercury reduction tube, after which the vapor is collected by a gold amalgamation tube; the tube then releases the stored elemental mercury vapor upon rapid heating, and the liberated vapor is subsequently detected using a cold atomic absorption spectrometer (CAAS) (Minervino et al., 2024),DMA enables direct sample injection for mercury determination without the need for sample digestion. This technology exhibits simple operation, rapid analysis, and minimal sample pretreatment requirements for blood mercury detection. DMA enables direct sample injection for mercury determination without the need for sample digestion. However, complex-matrix blood samples are prone to generating residues after high-temperature pyrolysis, which tend to adhere to the pyrolysis tubes and gold amalgamation collection tubes of the instrument. This induces a significant memory effect, where the preceding high-concentration sample contaminates subsequent low-concentration samples, resulting in falsely elevated detection results. In addition, such residue deposition impairs the performance of gold amalgamation collection tubes, leading to a reduced mercury atom adsorption capacity, an increased method detection limit, and compromised analytical precision (Sharma et al., 2020).

L- Cysteine is a naturally occurring thiol-containing chiral α-amino acid and a fundamental building block of proteins in organisms. Owing to its strong coordination ability with heavy metals via thiol groups, it is widely applied in heavy metal detection, biochemistry, and medicine (Han et al., 2019). Previous studies have demonstrated its efficacy as a mercury preservative and an ICP-MS cleaning agent (Wang et al., 2023). DL-Cysteine, a racemic mixture of L-cysteine and D-cysteine, also readily forms stable complexes with heavy metals, particularly mercury (Boroujerdi et al., 2016; Li et al., 1995). However, due to the relatively high cost of L-cysteine, we propose substituting it with the more cost-effective DL-cysteine to facilitate large-scale biomonitoring of mercury exposure in occupational populations. Therefore, this study aims to investigate whether the addition of DL-cysteine to the DMA can enhance the analytical performance of the mercury detection method to a certain extent.

In this study, we developed a novel approach by directly adding DL-cysteine into the sample boat. This interaction effectively mitigates sample carryover and memory effects, enabling sensitive, residue-free, rapid, and user-friendly determination of mercury in blood samples. Comparative experiments were conducted by incorporating DL-cysteine into blank measurements, calibration curve preparation, recovery assays, and precision tests, as well as by performing cross-instrument validation against alternative analytical platforms. The results demonstrated that the addition of DL-cysteine significantly enhanced the analytical performance of the direct mercury analyzer. Consequently, a new direct mercury determination method was successfully established, which exhibits excellent sensitivity, reproducibility, recovery, and anti-interference capability for blood mercury analysis. This method provides a promising new strategy for occupational health monitoring and clinical applications in mercury poisoning diagnosis.

## 2 Materials and methods materials and equipment

### 2.1 Materials

The DMA (MA-3000) was purchased from Nippon Instruments Corporation (Japan). DL-cysteine (Guaranteed reagent) was purchased from TianJing Berens Biotechnology Corporation (China). The relevant interference mixture was obtained from Weiye Measurement Corporation (China). AFS was obtained from obtabled Beijing Titan Instruments Corporation (China) was used for mercury determination.

### 2.2 Blood sample collection

Venous blood samples (3–5 mL) were collected using heparin anticoagulant tubes. Blank samples were prepared following the identical collection procedure. For transportation and storage, both blood and blank samples were placed in clean containers and maintained at 2 °C–8 °C.

### 2.3 Preparation of standard solution

According to the manufacturer’s instructions for the MA-3000 and relevant studies (Wang et al., 2023). The mercury single-element standard solution series 1 was prepared using a 0.1 g/L DL-cysteine solution and 2% nitric acid solution (Guaranteed reagent) as the dilution matrix, with concentration gradients of 0, 1, 5, 10, 20, 40, 80, and 100 μg/L. The mercury single-element standard solution series 2 was prepared using a 2% nitric acid solution as the dilution matrix, with concentration gradients of 0, 1, 5, 10, 20, 40, 80, and 100 μg/L. A 100 µL aliquot of each standard solution was transferred directly into the sample boat for analysis.

### 2.4 Analytical methodology and procedure

Prior to sample measurement, all sample boats were preheated under the instrumental conditions until a stable baseline was attained. For analysis, 100 µL of blood sample was precisely pipetted into a pre-conditioned boat, followed by the addition of 100 µL of DL-cysteine solution to ensure complete coverage of the sample. Mercury determination was performed using a DMA under the following optimized parameters: drying at 200 °C for 3 min; thermal decomposition with a temperature ramp to 650 °C over 1.5 min and holding for 2 min; reduction at 650 °C; amalgamation on a gold trap at 170 °C; rapid release of mercury from the amalgam at 900 °C for 30 s; and final detection by atomic absorption at 253.7 nm. The optimization of instrumental parameters has been supplemented, with details provided in the Supplementary Material.

### 2.5 Statistical analysis

All data were analyzed using GraphPad Prism version 9.0 (GraphPad Software, San Diego, CA, United States). The normality of continuous variables was assessed using the Shapiro-Wilk test. Continuous variables were presented as mean ± standard deviation (SD) or median and interquartile range (IQR), as appropriate. Differences between groups for continuous variables were compared using *Student’s* t-test (for normally distributed data) or the Mann-Whitney U test (for non-normally distributed data). A p-value <0.05 was considered statistically significant.

## 3 Result

### 3.1 Blank value determination

To investigate whether DL-cysteine could reduce the mercury background levels remaining in the sample boat, this study compared the background values of the sample boat with and without the addition of 100 μL DL-cysteine. The comparison was conducted after completing the test of a 100 μL blood sample containing 100 μg/L mercury and thoroughly cleaning the sample boat. The results showed that the baseline background value of the sample boat with 0.1 g/L DL-cysteine added was significantly lower than that of the non-addition group (Table 1).

**TABLE 1:** Comparison of background signals with and without the addition of DL-cysteine in the standard curve preservation solution.

Condition	Measured concentration (μg/L)									Mean concentration (μg/L)
	1	3	4	5	6	7	8	9	10	
0.1 g/L DL-cysteine	0.13	0.1	0.1	0.1	0.09	0.06	0.06	0.06	0.13	0.09
Without DL-cysteine	0.19	0.21	0.18	0.16	0.14	0.38	0.46	0.45	0.19	0.26

### 3.2 Sensitivity evaluation of DL-cysteine-assisted DMA method

**FIGURE 1:**
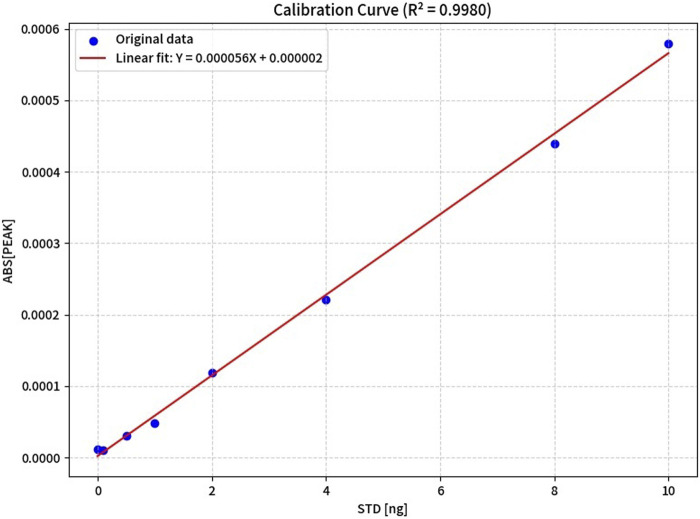
Calibration curves of mercury without DL-cysteine in sample boats.

In the experiment for establishing a mercury single-element standard curve, an experimental group supplemented with 0.1 g/L cysteine and a control group without cysteine were set up (Table 2). The results showed that for mercury single-element standard solution series 1 (with 0.1 g/L cysteine), the mercury mass concentration in the range of 0.0–100 μg/L exhibited a linear relationship with the corresponding ratio of signal intensity to that of the internal standard element. The linear regression equation was y = 0.0238x+0.0062, with a correlation coefficient (*R* ^2^) of 0.9996. The limit of detection was 0.5 μg/L, and the limit of quantification was 1.5 μg/L. For mercury single-element standard solution series 2 (without DL-cysteine): The mercury mass concentration in the range of 0.0–100 μg/L also showed a linear relationship with the corresponding signal ratio. The linear regression equation was Y=0.000056X + 0.000002, with a correlation coefficient (*R* ^2^) of 0.9980. The limit of detection was 0.9 μg/L, and the limit of quantification was 1.7 μg/L y=1.036x+6.142 is Figure 1.

**TABLE 2:** Mercury calibration curves with and without DL-Cysteine in sample boats.

Condition	Calibration curves	Correction coefficient R2	Concentration range (μg/L)	LOD (μg/L)	LOQ (μg/L)
0.1 g/L DL-cysteine	y = 0.0238x+0.0062	*R* ^2^ = 0.9996	0.0∼100	0.5	1.5
Without DL-cysteine	Y=0.000056X+0.000002	*R* ^2^ = 0.9980	0.0∼100	0.9	1.7

### 3.3 Repeatability assessment

To assess precision, standard solutions at three concentrations (20.0, 50.0, and 100.0 μg/L) were prepared in parallel, with six replicates analyzed per concentration within a single batch. A comparative experiment was performed whereby 100 μL of a 0.1 g/L DL-cysteine solution was added to the sample boat for one set of replicates, while the other set was left untreated. All determinations were strictly referenced to the same calibration curve (Table 3). The results demonstrated that at all concentration levels, the relative standard deviations (RSDs) of the DL-cysteine-treated group were consistently lower than those of the control group. This indicated that DL-cysteine exerted a certain stabilizing effect.

**TABLE 3:** Repeatability analysis for mercury determination with and without DL-cysteine addition in the sample boat.

0.1 g/L DL-cysteine			Without DL-cysteine		
Theoretical concentration (μg/L)	Mean concentration (μg/L)	RSD (%)	Theoretical concentration (μg/L)	Mean concentration (μg/L)	RSD (%)
20	19.58	3.28%	20	21.46	4.22%
50	50.65	1.76%	50	48.64	2.22%
100	98.13	0.73%	100	92.63	1.07%

### 3.4 Recovery assessment

The accuracy of the method was evaluated via spike recovery experiments. Samples at different concentration levels were selected, and spike recovery assays were conducted at three concentration gradients. The results showed that for samples of varying concentrations, the spike recovery rate for the group with DL-cysteine added to the sample boat ranged from 99.14% to 99.92%, while the recovery rate for the non-addition group was only 92.63%–97.58% (Table 4). This indicated that the addition of DL-cysteine to the sample boat could significantly improve the recovery of the target substance.

**TABLE 4:** Recovery analysis for mercury determination with and without DL-cysteine additio**n** in the sample boat.

Theoretical concentration (μg/L)	0.1 g/L DL-cysteine	Without-cysteine
​	Recover rate (%)	Recover rate (%)
20	99.14%	92.63%
50	99.29%	96,41%
100	99.92%	97.58%

### 3.5 Performance analysis of MeHg determination

This study was conducted to evaluate whether DMA could undergo complete thermal decomposition, be reduced to elemental mercury vapor, and release the collected elemental mercury for subsequent quantitative analysis via cold vapor atomic absorption spectrometry. A standard curve for MeHg was prepared by gravimetric dilution of a MeHg standard solution with 0.1 g/L DL-cysteine, yielding a series of concentrations at 0, 1, 5, 10, 20, 40, 80, and 100 μg/L. The calibration curve was plotted by correlating the signal values derived from the standard series with their corresponding mercury concentrations. Under the optimized experimental conditions, mercury in the solution was quantified, and the calibration curve was constructed based on the signal responses of the standard series against concentrations. The linear regression equation was determined as y = 0.0216x+0.0009, with a high correlation coefficient of *R* ^2^ = 0.9999 (Table 5). Additionally, the spiked recoveries for MeHg detection, with the addition of 0.1 g/L DL-cysteine to the sample boat, were in the range of 91.11%–98.97%.

**TABLE 5:** Recovery analysis of the MeHg determination.

Theoretical concentration (μg/L)	Determined concentration (μg/L)						Mean concentration (μg/L)	Recovery rate (%)
​	1	2	3	4	5	6	​	​
5	4.83	5.25	5.01	4.92	4.85	4.83	4.95	98.97%
20	18.61	17.82	18.22	18.17	18.11	18.4	18.22	91.11%
80	78.17	76.22	78.78	77.13	78.01	78.19	77.75	97.19%

### 3.6 Performance analysis of ethylmercury determination

A calibration curve for ethylmercury (EtHg) was established. An EtHg standard solution was gravimetrically diluted with 0.1 g/L DL-cysteine to prepare a standard series at concentrations of 0, 1, 5, 10, 20, 40, 80, and 100 μg/L. The calibration curve was plotted by plotting the signal values obtained from the standard solutions against their corresponding mercury concentrations. Mercury content in the test solutions was determined under the optimized experimental conditions, and the linear regression equation of the calibration curve (signal response vs. concentration) was calculated as y = 0.0188x+0.0008, with a correlation coefficient (*R* ^2^) of 0.9998 (Table 6). Furthermore, the spiked recovery rates for EtHg detection were in the range of 91.40%–97.65% upon the addition of 0.1 g/L DL-cysteine to the sample boat.

**TABLE 6:** Recovery analysis of the EtHg determination.

Theoretical concentration (μg/L)	Determined concentration (μg/L)						Mean concentration (μg/L)	Recovery rate (%)
​	1	2	3	4	5	6	​	​
5	4.61	4.35	4.51	4.47	4.61	4.87	4.57	91.40%
20	18.81	18.99	18.43	18.67	18.91	19.32	18.86	94.28%
80	78.23	77.11	79.22	77.42	78.15	78.57	78.12	97.65%

### 3.7 Performance analysis of total mercury

Mercury in the solution was determined under the established conditions, and a calibration curve was plotted using the signal values obtained from the standard concentration series versus the corresponding mercury concentrations. Under identical conditions, total mercury, MeHg, and EtHg were simultaneously measured. The correlation among the three was presented in Table 7. As shown in the chart, under the same calibration conditions, the results for total mercury, MeHg, and EtHg showed good consistency.

**TABLE 7:** Recovery analysis of the total mercury.

No.	Total mercury	MeHg	EtHg	Total/meHg CV	Total/EtHg CV
1	17.68	17.33	17.28	0.02	0.02
2	43.82	42.23	42.29	0.04	0.04
3	59.91	58.92	57.98	0.02	0.03
4	80.19	78.26	77.34	0.02	0.04

### 3.8 Interference analysis

Prepare a 20 ug/L mercury application solution and measure according to this method. To investigate whether DL-cysteine can mitigate the interference effects of different elements on the target detection system, this experiment was divided into two major groups: the experimental group supplemented with DL-cysteine and the control group without DL-cysteine supplementation. Within each of the two groups, one interference-free control subgroup and five single-element interference subgroups (selenium (Se), lead (Pb), arsenic (As), cadmium (Cd), and manganese (Mn); the multi-element mixture including copper, zinc, iron, calcium, magnesium) were set up, respectively. As shown in the table, the deviation of the test results is less than 10%, indicating that the concentrations of Se, Pb, As, Cd, Mn, etc., Have no interference with the test results at the 100 ug/L level. As shown in Table 8 below, the presence or absence of DL-cysteine in the sample boat did not materially affect the outcome of the interference resistance tests.

**TABLE 8:** Interference analysis of mercury determination with and without DL-cysteine addition in the sample boat.

With DL-cysteine	Mean concentration (μg/L)	SD (%)	Without DL-cysteine	Mean concentration (μg/L)	SD (%)
Without interference	20.1	—	Without interference	20.1	—
100ug/L Se	20.4	1.49%	100ug/L Se	19.21	−1.42%
100ug/L Pb	19.7	−1.99%	100ug/L Pb	20.07	1.11%
100ug/L As	19.8	−1.49%	100ug/L As	19.23	−1.19%
100ug/L Cd	20.5	1.99%	100ug/L Cd	19.87	1.68%
100ug/L Mn	20.7	2.98%	100ug/L Mn	20.62	1.96%

### 3.9 Sample stability assessment

The same blood sample was used to prepare two sets of test solutions: one with 0.1 g/L DL-cysteine added to the sample boat and the other without. Each set was analyzed in ten parallel measurements. The average concentration and relative standard deviation (RSD) were calculated, and the results are presented in Table 9. In the actual sample matrix, the group with DL-cysteine addition showed a lower RSD than the group without DL-cysteine, indicating that the method is robust and suitable for accurate quantitative analysis of real samples.

**TABLE 9:** Stability analysis of mercury determination with and without DL-cysteine addition in the sample boat.

Condition	Determined concentration (μg/L)									Mean concentration (μg/L)	RSD (%)
​	1	2	3	4	5	6	7	8	9	​	​
Without DL-cysteine	54. 57	59.02	56.6	57.78	55.67	56.64	55.94	58. 42	54.53	56.60	2.58%
0.1 g/L DL-cysteine	58. 84	57.7	57.49	56.26	58.16	56.5	57.52	57.59	58.71	57.49	1.39%

To investigate whether the duration of residence in the sample boat affects the sample results, this study selected blood samples at the same concentration level. The same blood sample was divided into two groups, each consisting of 20 subsamples. In one group, DL-cysteine was added to the sample boat, while no addition was made in the other group. The samples were analyzed sequentially in the sample boat in order of introduction to study whether the residence time in the sample boat influences the experimental outcomes. The results showed that for samples at the same concentration, the degradation rate for those with added DL-cysteine was 5.61%, compared to 8.83% for those without DL-cysteine. This indicates that the addition of DL-cysteine reduces the degradation rate to some extent (Table 10).

**TABLE 10:** Loss rate analysis of analysis of mercury determination with and without DL-cysteine addition in the sample boat.

Condition	1 h Declin rate (%)	2 h Declin rate (%)	4 h Declin rate (%)	6 h Declin rate (%)
Without DL-cysteine	2.53%	3.99%	6.91%	8.83%
0.1 g/L DL-cysteine	1.76%	2.29%	3.92%	5.61%

### 3.10 Comparison with AFS

To compare the performance of the 0.1 g/L DL-cysteine-modified DMA and AFS for the same samples, both methods were used for analysis. Results showed slightly higher detection values with the modified DMA method than with AFS (Table 11). This is likely due to the elimination of sample digestion (reducing mercury loss) and the residue-mitigating effect of DL-cysteine, which further minimizes mercury loss during detection.

**TABLE 11:** Comparison experiment.

Condition	1	2	3	4	5	6
DMA	10.296	18.801	28.2	43.065	49.883	51.306
AFS	10.34	18.96	28.52	45.55	49.96	51.61

### 3.11 Practical application

**FIGURE 2:**
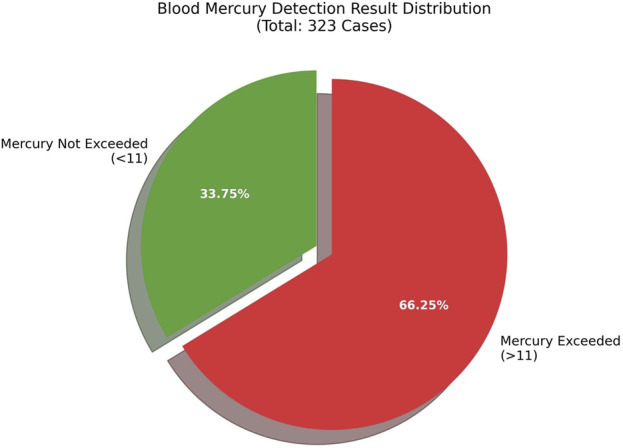
The proportion of blood mercury levels exceeding the threshold in 2025.

In 2025, the DMA developed in this study was applied to analyze 323 biological samples. A total of 124 individuals exhibited blood mercury levels exceeding the normal threshold, consistent with expected background exposure (Figure 2). Our hospital undertakes long-term mercury exposure monitoring for some occupational groups. For patients found to have excessive mercury levels during monitoring, a scientific and standardized comprehensive treatment plan was adopted. After systematic treatment, the patients’ conditions were effectively controlled and gradually improved.

## 4 Discussion

Mercury is a global pollutant characterized by high toxicity and persistence. It readily bioaccumulates in organisms, posing a severe threat to human health and the environment, particularly for occupational populations with mercury exposure (Suen et al., 2025). Therefore, the rapid, convenient, and accurate determination of mercury in blood samples is crucial for the diagnosis and treatment of mercury-related diseases. Guizhou Province of China is rich in mercury ore deposits and hosts large-scale mercury mining and processing activities. Consequently, certain occupational groups in this region are chronically exposed to hazardous mercury-contaminated environments (Wang et al., 2023). Thus, developing a highly efficient, simple, and rapid detection method is of particular importance for this study. DMA enables the direct determination of mercury without the need for sample digestion. This technique offers simple operation, rapid analysis, and minimal requirements for sample pretreatment in blood mercury detection. DL-cysteine can form stable complexes with heavy metals, especially mercury, acting as an effective mercury stabilizer.

The results of the blank value experiments indicated that the addition of DL-cysteine significantly reduced the background signal of the detection system. This phenomenon confirms its effectiveness in mitigating the adsorption of mercury during analysis and inhibiting the accumulation of mercury residues on the surface of the sample boat. Based on this, it can be inferred that the mechanism by which DL-cysteine improves residue issues is primarily attributed to its ability to stabilize mercury species and inhibit mercury loss. DL-cysteine molecules can form stable complexes with mercury ions, a structure that effectively prevents mercury loss caused by adsorption onto container walls, volatilization, or co-precipitation with the sample matrix (Rujiwatra et al., 2022; Wang et al., 2016).

During the establishment of the standard curve, the standard solutions containing DL-cysteine exhibited a superior analytical response, suggesting that DL-cysteine exerts a stabilizing and protective effect. According to relevant studies, many analysts use gold, other compounds such as L-cysteine, disodium hydrogen phosphate, potassium bromide, sodium sulfide, dithiothreitol, ethylenediaminetetraacetic acid, and 2-mercaptoethanol as the stabilizer for mercury to enhance the analytical performance (Chai et al., 2006; Jin et al., 2017). In our study, the method fully demonstrates that DL-cysteine can achieve stabilization effects comparable to those of the aforementioned substances (Jin et al., 2017).

Validation experiments for repeatability, stability, and recovery further confirmed that the experimental group with DL-cysteine supplementation exhibited superior repeatability and stability in the detection results. The performance of direct mercury analyser (DMA) for blood mercury detection. We propose that its mechanism of action stems from the ability of DL-cysteine to form stable complexes with mercury, thereby reducing the inherent tendency of mercury to adsorb onto the surfaces of glass, plastic containers, and instrument injection tubes. This, in turn, alleviates the adsorption effect and memory effect to a certain extent, ensuring the reliability of continuous batch analysis (Drebushchakv et al., 2008).

In the experimental analysis of organic mercury, the detection of both methylmercury and ethylmercury exhibited excellent analytical performance following the addition of DL-cysteine, with no significant difference observed compared with that of elemental mercury. In studies related to mercury poisoning, organic mercury is recognized as a major contributor to intoxication incidents (Susa et al., 2023)). Based on this, this study focused on investigating whether DMA can completely convert organic mercury in samples into elemental mercury vapor without complex pretreatment, thereby enabling the accurate determination of total mercury content. The experimental results confirmed that, within the DMA detection system, organic mercury undergoes complete thermal decomposition and is reduced to elemental mercury vapor. The detection results were consistent with those of other mercury species (e.g., inorganic mercury). This finding fully validates the accuracy and reliability of the DL-cysteine-assisted DMA method established in this study for blood mercury analysis, ensuring that the method can truly reflect the total mercury level in blood samples. Furthermore, this study it lacks mercury speciation capability and cannot identify or quantify specific forms such as organic and inorganic mercury in samples. Therefore, future research can focus on optimizing mercury speciation detection technologies to further improve the blood mercury analysis system, thereby providing more robust technical support for the accurate diagnosis and etiological analysis of mercury poisoning.

The inherent technical advantages of DMA, such as short detection time and simple operation, further enhance its practicability in blood mercury analysis and provide an excellent detection method for the rapid testing of large-scale samples.This method eliminates the need for traditional sample pretreatments such as microwave digestion or wet digestion. This characteristic not only reduces the requirement for highly skilled operators and makes it well-suited for primary laboratories such as community hospitals Blood samples can be directly introduced into the instrument, obviating cumbersome procedures like acid digestion, acid evaporation, and volume adjustment. The total analysis time per sample, from injection to result output, is only 8–10 min. In contrast, CVAAS and AFS typically require approximately 30 min (including pretreatment), while ICP-MS takes about 20 min. This high throughput enables the analysis of hundreds of blood samples daily, making it highly suitable for large-scale screening scenarios such as occupational health surveillance and clinical batch testing.

## 5 Conclusion

A DL-cysteine-based DMA method for the determination of blood mercury was established in this study. DL-cysteine was incorporated at three key stages: it was added to the blank solution during background measurement, used as a mercury preservative in the preparation of the standard curve, and introduced into the sample boat for the precision, recovery, and blood sample testing experiments. The results demonstrated that the incorporation of DL-cysteine yielded a MDL of 0.5 μg/L and a LOQ of 1.5 μg/L. The method precision ranged from 0.73% to 3.28%, with spike recovery rates between 99.14% and 99.92%. In actual sample analysis, the method exhibited robust repeatability and stability. Through the stabilizing and complexing effects of DL-cysteine, the accuracy, precision, repeatability, and stability of the detection method were effectively enhanced, with all analytical metrics meeting the requirements for the quantitative analysis of blood mercury. Furthermore, this study confirmed that DMA can completely convert organic mercury in samples into elemental mercury vapor without the need for complex sample pretreatment, thereby ensuring the accuracy and reliability of total mercury determination. This method offers the advantages of high efficiency, convenience, safety, and low cost. It is suitable for the routine monitoring of blood mercury levels in occupationally exposed populations and can provide reliable technical support for occupational health protection, as well as the early screening and clinical diagnosis of mercury poisoning.

## Data Availability

The original contributions presented in the study are included in the article/Supplementary Material, further inquiries can be directed to the corresponding author.
