# Supplementary material for: Quantitative determination of mercury in blood-based DL-cysteine-modified direct mercury analyzer method
Source: Front Bioeng Biotechnol. 2026 Mar 12;14:1776977. doi: 10.3389/fbioe.2026.1776977 (PMC13017820; doi:10.3389/fbioe.2026.1776977)
Supplement: Supplementary file 1 [file Supplementaryfile1.pdf]

Supplementary Material

1. The optimization of instrumental parameters

1.1 Selection of Drying Temperature

A blood sample with a concentration of 30.0 μg/L was prepared. An aliquot of 100 μL (same volume for each test) was injected, and the spiked recovery rate was determined under different drying temperatures. As shown in Table 12, the spiked recovery rate was relatively the highest at 200 °C. Therefore, 200 °C was finally selected as the optimal drying temperature.

Table 1 Effects of Different Drying Temperatures on Spiked Recovery Rate

Drying Temperature (℃)	1st Test	2 nd Test	3 rd Test	Average Value
150	96.39%	97.33%	98.00%	97.24%
200	98.56%	97.11%	98.28%	97.98%
250	95.89%	96.39%	96.55%	96.27%
300	96.44%	95.83%	95.50%	95.92%

1.2 Selection of Drying Time

A blood sample with a concentration of 30.0 μg/L was prepared. An aliquot of 100 μL (the same volume for each test) was injected, and the changes in spiked recovery rate were determined under different drying times. As shown in Table 13, the spiked recovery rate reached the highest value of 99.39% at 3 minutes. Therefore, 3 minutes was finally selected as the optimal drying time.

Table 2 Effects of Different Drying Times on the Determination of Spiked Recovery Rate

Drying Time（min）	1st Test	2 nd Test	3 rd Test	Average Value
0	97.78%	98.39%	97.50%	97.88%
2	99.28%	98.72%	98.17%	98.72%
3	99.34%	99.00%	99.78%	99.39%
4	98.78%	98.39%	99.22%	98.78%
5	97.89%	98.22%	97.67%	97.94%

1.3 Selection of Ashing Temperature

A blood sample with a concentration of 30.0 μg/L was prepared. An aliquot of 100 μL (the same volume for each test) was injected, and the changes in spiked recovery rate were determined under different ashing temperatures. Considering the characteristics of actual samples and the average spiked recovery rate, as shown in Table 14, the spiked recovery rate reached the highest value of 98.95% at 650 °C. Therefore, 650 °C was finally selected as the optimal ashing temperature.

Table 3 Effects of Different Ashing Temperatures on the Determination of Spiked Recovery Rate

Ashing Temperature (°C)	1st Test	2 nd Test	3 rd Test	Average Value
120	98.11%	97.22%	97.67%	97.67%
180	97.44%	98.28%	97.89%	97.89%
300	97.55%	97.78%	98.17%	97.83%
550	98.00%	98.61%	98.45%	98.33%
650	99.17%	98.39%	99.22%	98.95%
800	97.89%	97.61%	98.95%	98.17%

1.4 Selection of Ashing Time

A blood sample with a concentration of 30.0 μg/L was prepared. An aliquot of 100 μL (the same volume for each test) was injected, and the changes in spiked recovery rate were determined under different ashing times. Considering the characteristics of actual samples and the average spiked recovery rate, as shown in Table 15, the spiked recovery rate reached the highest value of 97.55% at 2 minutes. Therefore, 2 minutes was finally selected as the optimal ashing time.

Table 4 Effects of Different Ashing Times on Spiked Recovery Rate

Ashing Time（min）	1st Test	2 nd Test	3 rd Test	Average Value
0.5	97.00%	96.94%	96.66%	96.89%
1	97.55%	96.39%	97.89%	97.28%
2	97.28%	97.72%	97.67%	97.55%
3	97.44%	97.33%	97.72%	97.50%
4	97.83%	97.17%	96.55%	97.17%
